# Gr/gr deletions on Y-chromosome correlate with male infertility: an original study, meta-analyses, and trial sequential analyses

**DOI:** 10.1038/srep19798

**Published:** 2016-02-15

**Authors:** Sandeep Kumar Bansal, Deepika Jaiswal, Nishi Gupta, Kiran Singh, Rima Dada, Satya Narayan Sankhwar, Gopal Gupta, Singh Rajender

**Affiliations:** 1 Division of Endocrinology, Central Drug Research Institute, Lucknow, UP, India; 2 Department of Molecular and Human Genetics, Banaras Hindu University, Varanasi, UP, India; 3 Lab for Molecular Reproduction and Genetics, All India Institute of Medical Sciences, New Delhi, India; 4 Departmentof Urology, King George’s Medical University, Lucknow, India

## Abstract

We analyzed the AZFc region of the Y-chromosome for complete (b2/b4) and distinct partial deletions (gr/gr, b1/b3, b2/b3) in 822 infertile and 225 proven fertile men. We observed complete AZFc deletions in 0.97% and partial deletions in 6.20% of the cases. Among partial deletions, the frequency of gr/gr deletions was the highest (5.84%). The comparison of partial deletion data between cases and controls suggested a significant association of the gr/gr deletions with infertility (P = 0.0004); however, the other partial deletions did not correlate with infertility. In cohort analysis, men with gr/gr deletions had a relatively poor sperm count (54.20 ± 57.45 million/ml) in comparison to those without deletions (72.49 ± 60.06), though the difference was not statistically significant (p = 0.071). Meta-analysis also suggested that gr/gr deletions are significantly associated with male infertility risk (OR = 1.821, 95% CI = 1.39–2.37, p = 0.000). We also performed trial sequential analyses that strengthened the evidence for an overall significant association of gr/gr deletions with the risk of male infertility. Another meta-analysis suggested a significant association of the gr/gr deletions with low sperm count. In conclusion, the gr/gr deletions show a strong correlation with male infertility risk and low sperm count, particularly in the Caucasian populations.

Y-chromosome partial deletions, leading to the removal of male specific genes, constitute an important etiological factor in male infertility^1^. Approximately, 25–55% of the patients with severe testicular pathologies (hypospermatogenesis, sperm maturation arrest and Sertoli cell only syndrome) and 5–25% of the patients with severe oligozoospermia or azoospermia harbor these deletions, making them the most common known genetic cause of spermatogenic failure^2^,^3^,^4^,^5^,^6^,^7^,^8^. These deletions occur in three non-overlapping regions (AZFb and AZFc are partially overlapped) mapped to the proximal (AZFa), middle (AZFb), and distal (AZFc) portions of the Y-chromosome^9^. Deletions of AZFa lead to the complete depletion of germ cells^5^ and that of AZFb result in spermatogenic arrest^10^, but both these deletions are less frequent. Deletions in the AZFc region are more frequent and result in variable outcomes from mild to severe spermatogenic failure that may be compatible with natural conception or assisted reproduction^5^,^11^,^12^,^13^. The AZFc region is comprised of repeated sequences and is most vulnerable to deletions. A b2/b4 deletion, spanning 3.5 Mb region, removes the complete AZFc region, which contains twelve genes and transcriptional units in multiple copy numbers^14^. Among all Y microdeletions, AZFc deletions are the most frequent (~80%)^10^, followed by AZFa (0.5–4%) and AZFb (1–5%) deletions. In addition to the large deletions, partial deletions such as gr/gr (1.6 Mb), b1/b3 (1.6 Mb), b2/b3 (1.8 Mb), occurring within the AZFc region have been identified using non-repeated STS markers^10^,^14^,^15^,^16^,^17^.

Among partial deletions, gr/gr are the most frequent and major factor contributing to male infertility. Repping *et al.* (2003) reported that gr/gr deletions do not completely eliminate any testis specific gene family, but reduces the copy number of gene families on the Y-chromosome^14^. They also observed that the dosage of one or more of these families affects the quality of sperm produced. Therefore, the semen picture in gr/gr deletions may vary from azoospermia to normozoospermia and can differ ethnically and geographically. Several studies have reported different frequencies of gr/gr deletions with contradictory outcomes regarding their correlation with male infertility. A number of studies have reported a significant association between the deletions and spermatogenic failure^18^,^19^,^20^,^21^,^22^; however, an almost equal number of studies has suggested a lack of any such association^23^,^24^,^25^,^26^,^27^,^28^. Y haplotype is one of the factors contributing to such variations; for example, the gr/gr deletions have been suggested to be a risk factor only in the populations where these deletions are not fixed^29^. Other partial deletions (b2/b3 and b1/b3) in the AZFc region are rare and have been studied less often.

The presence of multiple genes/gene copies on the Y-chromosome suggests a quantitative relationship between the gene dosage and spermatogenesis^30^. Further, the classification of subjects into cases and controls on the basis of fertility status does not take sperm count into consideration. Since fertility and normozoospermia are not synonymous, a few recent studies have recommended a cohort analysis to better assess the correlation between Y-partial deletions and sperm count/semen quality^21^,^30^,^31^. Therefore, we designed the present study to find the frequency of complete (b2/b4) and partial (gr/gr/, b1/b3, b2/b3) AZFc deletions in Indian populations for their association with spermatogenic failure/male infertility. Further, we also performed meta-analyses and trial sequential analyses to estimate the impact of the gr/gr deletions on sperm count and male fertility.

## Materials and Methods

### Study Group

The study was approved by the Institutional Ethics Committee of the Central Drug Research Institute and that of the King George’s Medical University, Lucknow. The experiments were carried in accordance with the guidelines approved for research on human samples. We enrolled participants after obtaining their informed written consents. All the subjects belonged to the Indo-European ethnicity. Male infertility was defined as the inability to initiate a pregnancy after one year or more of regular unprotected intercourse. Before sample collection, the subjects underwent detailed medical and physical examinations. The possibility of involvement of female factors was ruled out before enrollment of a patient in the study. Further, the patients with endocrine abnormalities (hypogonadism) and those with gross dysmorphic abnormalities, acquired and congenital structural defects of the urogenital system (cystic fibrosis, Young’s syndrome, etc.), were excluded. The patients with the history of surgical intervention of genital tract obstruction/dysfunction (varicocele, obstructive azoospermia) were also excluded. The infertile individuals used to excessive alcohol consumption, drug abuse (ecstasy, marijuana and recreational substances) and those having been exposed to radiations as a part of radiotherapy, were also excluded. The patients were evaluated for karyotype abnormalities, and those showing chromosomal abnormalities were excluded. The patient group, after following the above inclusion and exclusion criteria, comprised of 822 idiopathic infertile individuals.

Semen samples were collected by masturbation after 3–5 days of abstinence. Semen volume was recorded after liquefaction, followed by the assessment of sperm count and motility using computer assisted sperm analyzer (CASA). Semen parameters were investigated according to the World Health Organization (WHO) criteria, (2010)^32^. The patients were classified into azoospermic (absence of mature sperm in semen, n = 251), oligozoospermic (sperm count: <15 million/ml, n = 105), normozoospermic (sperm count: ≥15 million/ml, n = 203), asthenozoospermic (progressive sperm motility: <32% and sperm count: ≥15 million/ml, n = 34), and uncategorized idiopathic infertile individuals (patients who did not provide semen samples, but female factor infertility had been ruled out, n = 229). The diagnosis of azoospermia was established by pellet analysis after semen centrifugation that was repeated at least twice to confirm azoospermia. We also collected five ml blood samples from the patients for the purpose of DNA isolation. Case-control analysis was done on all cases (n = 822), irrespective of the availability of semen picture while cohort analysis was done for only 342 patients as 251 were azoospermic and semen details of 229 were not available.

For comparison purpose, 225 proven fertile men were recruited as controls. The control samples were taken from volunteers visiting the clinic for problems other than infertility. All control individuals had fathered at least one child during the last three years and never had any sexual abnormality. The controls belonged to the same age-group (20–45 years) and had the same ethnicity as that of the patients. Semen samples of the control individuals were not collected; hence, their fertility was ascertained by the information provided by them. Due to the lack of semen parameters for this group, the control samples were not included in the cohort analysis. This group served the purpose of a reference group for case-control analysis only.

### Deletion screening

Genomic DNA was isolated from the peripheral blood samples following the protocol described in our earlier study^33^. Y-chromosome partial deletions were analyzed using five sequence-tagged site (STS) markers (sY1161, sY1191, sY1291, sY1206 and sY1201) as described in previous studies^14^,^17^,^34^. The locations of the STS markers and the respective deletions on the Y-chromosome are shown in the supplementary Figure S1. Details of the primer sequences used for amplification and the corresponding PCR conditions have been summarized in the supplementary Table S1. Y-chromosome partial deletions were detected upon failure of amplification of a particular STS marker/set of markers (supplementary Table S2). All PCR reactions were performed in a simplex reaction with SRY as a control (amplification of the *SRY* gene was checked for all samples as a simplex PCR reaction). At least one repetition for the amplified markers and two repetitions for the failed markers were carried out to confirm the genotypes. Deletions confirmed upon these investigations were considered to be the true deletions.

### Statistical analysis

All statistical tests were carried out using the biostatistical tools available online (www.vassarstats.net and http://www.graphpad.com/quickcalcs/). Statistical tests were repeated at least twice to confirm the results. P values less than 0.05 were considered as statistically significant difference.

#### Cohort analysis

For cohort analysis, the patients were classified into gr/gr deleted and non-deleted groups. The average of semen parameters was compared between these groups using ‘t’ test. We did not take into account the data for azoospermic patients as they had no contribution to the semen parameters and their inclusion would have biased the results. Since partial deletions were hypothesized to compromise semen quality, one tailed data analysis was carried out.

#### Case-control analysis

For case-control analysis, the difference in the frequencies of gr/gr deletions was compared between patients and controls using the Fisher’s exact test.

### Meta-analysis

A number of studies have analyzed the gr/gr deletions in infertile and fertile men of different geographic and ethnic populations. These studies have presented contrasting results regarding the correlation of gr/gr deletions with male infertility and their clinical significance. Therefore, we undertook meta-analyses by pooling all published data that fulfilled a well defined inclusion criteria in order to build a consensus regarding the correlation of gr/gr deletions with male infertility and sperm count.

#### Identification of studies

A systematic literature search in the public databases; ‘Pubmed’, ‘Google Scholar’ and ‘ScienceDirect’, was conducted using the keywords; ‘AZFc deletions’, ‘gr/gr deletions’, ‘male infertility’, ‘Y-partial deletions’ and ‘Y-subdeletions’, in various combinations. The irrelevant studies were excluded by reading the abstracts of articles. Full text articles of all relevant studies were collected from the respective journals or from the authors via e-mail. Citations in these papers were looked carefully to identify other relevant studies. The studies thus selected were further subjected to inclusion and exclusion criteria, followed by data extraction from the shortlisted articles. Further, we categorized all the studies on the basis of geography and ethnicity and performed subgroup meta-analyses. Studies from China, Japan, Korea and nearby regions were classified under the Mongolian sub-group while studies from Europe and nearby regions or those with a Caucasian appearance were classified under the Caucasian sub-group. Studies from America and nearby regions as well as those stating a mixed nature of the subjects were classified under the mixed ethnicity sub-group. Two studies from North Africa (Tunisia and Morocco) were classified as Nigro-Caucasian because of a Nigro-Caucasian appearance of the subjects. Studies from south India and Sri Lanka were classified as Dravidians. Studies from North India were classified under the Caucasian sub-group because of their close ethnic affinity with the latter^35^. Further, we categorized the studies into the following regional sub-groups on the basis of geographic locations; America, Europe, East Asia (China, Japan, Korea and nearby regions), South Asia (India, Iran), South-East Asia (Malaysia) and North Africa.

To evaluate the effect of gr/gr deletions on sperm count, we carefully examined all the studies for availability of genotype and sperm count data. Most of the studies had not provided sperm count data for the control group. Therefore, only the studies providing sperm count data for the subjects (cases/controls) with and without deletions were included in this analysis, and mean, standard deviation and sample size of the case and control groups were extracted. Two studies^21^,^30^ had provided data in the median and range format; therefore, we estimated mean and standard deviation as described by Hozo *et al.* (2005)^36^. This way, a meta-analysis for comparison of sperm count between the gr/gr deleted and non-deleted groups was performed. For this, the mean difference (MD) and 95% confidence interval were chosen as effect sizes.

#### Inclusion criteria

Inclusion criteria for meta-analysis were:

1. Each trial was an independent case-control study.
2. Inclusion of the patients was done according to the standard diagnostic parameters.
3. The purpose of all the studies was similar.
4. The study had supplied enough information for the calculation of the odds ratio/mean difference.
5. Standard methods were used to analyze Y-partial deletions at high-resolution level.

#### Exclusion criteria

The studies not providing a detailed description of the subjects, raw data, and other information required to precisely understand the study design and the data therein were excluded.

#### Data extraction and statistical approach

Meta-analysis was conducted using the Comprehensive Meta-Analysis software (version-2). Dichotomous data in form of the number of individuals having gr/gr deletions in the case and control groups and the total sample size in each group was fed into the software. Odds ratio (OR) and 95% confidence interval were chosen as the effect sizes. For meta-analysis on sperm count, the mean difference (MD) and 95% confidence interval were chosen as the effect sizes.

The heterogeneity was assessed using Cochran ‘Q’ test. Since the ‘Q’ statistics gives an idea about the presence of heterogeneity qualitatively, I^2^ value was used to quantify the degree of heterogeneity between studies. I^2^ values suggested by Higgins and Thompson were used to infer about the magnitude of heterogeneity; viz. 25%, 50% and 75%, which correspond to low, medium and high heterogeneity, respectively^37^. Publication bias was evaluated using the funnel plot of standard error (std error vs log odds ratio) and the Egger’s regression test of significance. The recommended models for the absence of a significant heterogeneity (Mantel-Haenszel fixed effect model, Peto method) and the presence (DerSimonian-Laird random effects model, DL method) were used^38^,^39^. We also performed the sensitivity analysis by two methods. The first approach removed the studies based on small sample size (less than 100 in either of case/control groups) and the second approach removed one study at a time, followed by recalculation of estimates on the remaining studies.

#### Trial sequential analysis (TSA)

Outcome of meta-analysis is prone to systematic (bias) or random errors (play of chance) due to dispersed data and repeated significance testing. Bias from the trials with low methodological quality, publication bias and small trials may result in false p value. Therefore, we used trial sequential analysis (TSA) tool of the Copenhagen Trial Unit, Center for Clinical Intervention Research, Denmark, which calculates the required information size (number of samples) by adjusting the significance level for dispersed data and confirms statistical reliability of the data^40^. Some previous studies have shown that the outcome of TSA is more reliable than that of the traditional meta-analysis^41^,^42^. The required information size was calculated by considering an overall type–I error of 5% and type-II error of 20%. The TSA tool plotted a two-sided graph where red straight lines indicate the significance boundaries of traditional meta-analysis, the blue line shows cumulative Z-score, and red lines sloping inwards represent the trial sequential monitoring boundaries with adjusted p-values.

## Results

### Complete AZFc deletions occur only in the cases

Deletions of the complete AZFc region (b2/b4) were observed in the cases only (n = 8) (Table 1). Among the cases having complete AZFc deletion, four were azoospermic and four were uncategorized idiopathic infertile individuals. B2/b4 deletions were not observed in the normozoospermic infertile or fertile control individuals. Given the fact that b2/b4 is already established as a causative factor for infertility^43^,^44^ and that we did not find its occurrence in the control group, statistical comparison for the complete AZFc deletions was not undertaken. One case with a b2/b4 deletion also failed to confirm the presence of sY1161 marker, suggesting the extension of deletion beyond the AZFc region.

### Partial deletions in the AZFc region are relatively common

**Figure 1:**
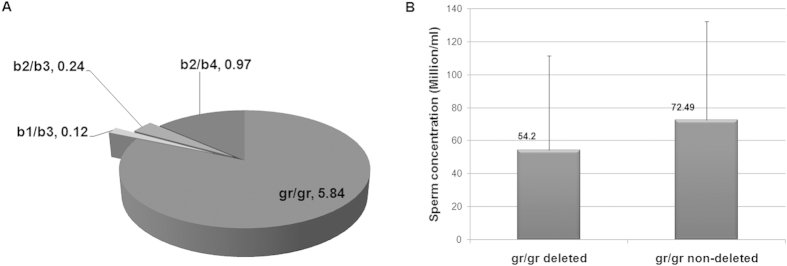
Distribution and the effect of Y-partial deletions: (**A**) Pie chart showing the frequencies (%) of the AZFc partial deletions in the cases (n = 822), (**B**) Cohort analysis showing sperm count between groups with and without gr/gr deletions.

We observed that 6.20% of all the cases (n = 822) had some or other partial deletion in the AZFc region (Table 1). Out of these, the percentage of individuals with gr/gr deletions was the highest, 5.84% (Fig.1A and Table 1). The gr/gr deletions showed more diversified presence with a significant frequency across almost all case sub-groups (except asthenozoospermic) with a minor frequency in the fertile control group. Other deletions were observed in lesser percentages that have been summarized in Fig. 1A. The control group (n = 225) had two individuals with gr/gr deletions and one with a b2/b3 deletion. No partial deletion was observed in the asthenozoospermic group (n = 34) (Table 1).

Among the cases harbouring partial deletions (n = 51), four were azoospermic, seven were oligozoospermic, eighteen were normozoospermic and twenty-two were uncategorized idiopathic infertile individuals (Table 1). b2/b3 deletion was seen in one azoospermic and one uncategorized idiopathic infertile individual while b1/b3 deletion was seen in one uncategorized idiopathic infertile individual only. One case lacked only sY1161 marker and four cases lacked only sY1201 marker, which suggested intact AZFc region, resulting in their classification as no-deletion cases. These cases may have deletions beyond the AZFc region, which is beyond the scope of this study.

### Case-control analysis suggests increased infertility risk in gr/gr deletion carriers

As mentioned above, gr/gr deletions were the most frequent among partial deletions that were followed by b2/b3 and b1/b3 deletions (Table 1). The comparison of gr/gr deletions frequency revealed a highly significant difference between cases and controls (p = 0.0004) with an increased risk of infertility in the deletion carriers (Table 2). However, the comparison of other partial deletions (b1/b3 and b2/b3) showed no significant association with infertility (Table 2).

### Cohort analysis shows relatively lesser sperm count in gr/gr deletions carriers

Looking at the diverse presence of gr/gr deletions across infertile and control groups, we undertook a cohort analysis for assessing the impact of these deletions on sperm count. A cohort of three hundred and forty two patients was classified into two groups on the basis of the deletion status [with deletions (n = 25) and without deletions (n = 317)], which was followed by the comparison of average sperm count between the two. Azoospermic and other patients, whose semen profiles were not available, were excluded from this analysis. We found that men with gr/gr deletions had a relatively lesser sperm count (sperm concentration: 54.20 ± 57.45 million/ml) in comparison to those without deletions (sperm concentration: 72.49 ± 60.06 million/ml), though the difference did not reach statistical significance (t statistics = 1.4704, df = 340, one tailed p value = 0.071 at 95% CI) (Fig.1B). Nevertheless, the sperm count in both these groups was within the normozoospermic range as per the WHO 2010 criteria.

### Literature search for meta-analysis

**Figure 2:**
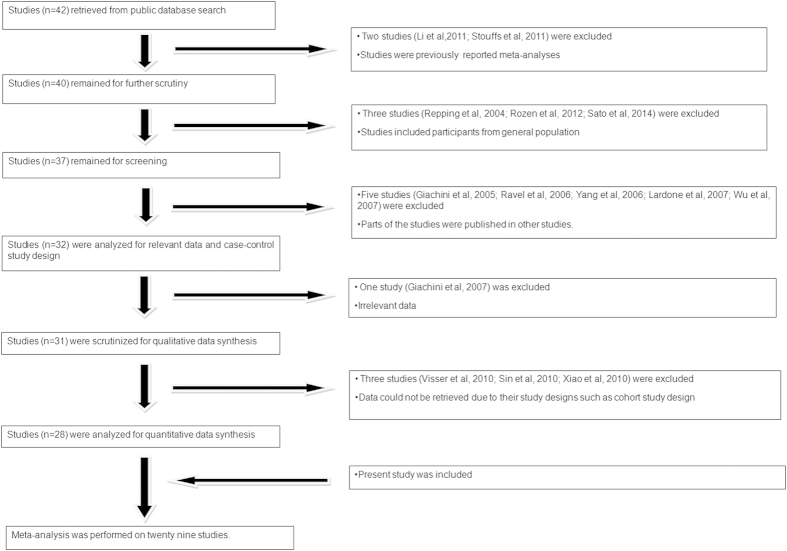
Retrieval of studies for meta-analysis: Flow diagram showing stepwise screening of the studies for inclusion in the meta-analysis.

Literature search retrieved forty-two studies, which were subjected to the inclusion criteria. Out of these, fourteen studies were excluded because of the following reasons: two studies were meta-analyses^45^,^46^, three had recruited participants from the general population^15^,^31^,^47^, parts of five studies had been published in other studies^23^,^48^,^49^,^50^,^51^, one study had irrelevant data^52^ and three studies had irrelevant study design^21^,^27^,^53^. After including the present case-control study, meta-analyses were performed on twenty-nine studies^14^,^18^,^19^,^20^,^22^,^28^,^30^,^54^,^55^,^56^,^57^,^58^,^59^,^60^,^61^,^62^,^63^,^64^,^65^,^66^,^67^,^68^,^69^,^70^,^71^,^72^,^73^,^74^ (Fig. 2 and supplementary Table S3).

### Quantitative data synthesis

This meta-analysis included twenty-nine studies consisting of 10948 cases and 6604 controls (supplementary Table S4). Five studies had less than a hundred samples in either of the study groups^56^,^58^,^66^,^67^,^69^. Eleven studies, including the present study, showed a significant association between the gr/gr deletions and male infertility^14^,^18^,^19^,^20^,^22^,^30^,^54^,^61^,^68^ while others showed no association. Very few studies were undertaken on Australian (n = 1)^19^, South-East Asian (n = 1)^69^, North African (n = 2)^28^,^59^, Dravidian-Indian (n = 2)^58^,^73^ and Nigro-Caucasian (n = 2)^28^,^59^ populations; therefore, sub-group meta-analysis on these regions/populations was not undertaken.

### Test of significance and heterogeneity by conventional meta-analysis

**Figure 3:**
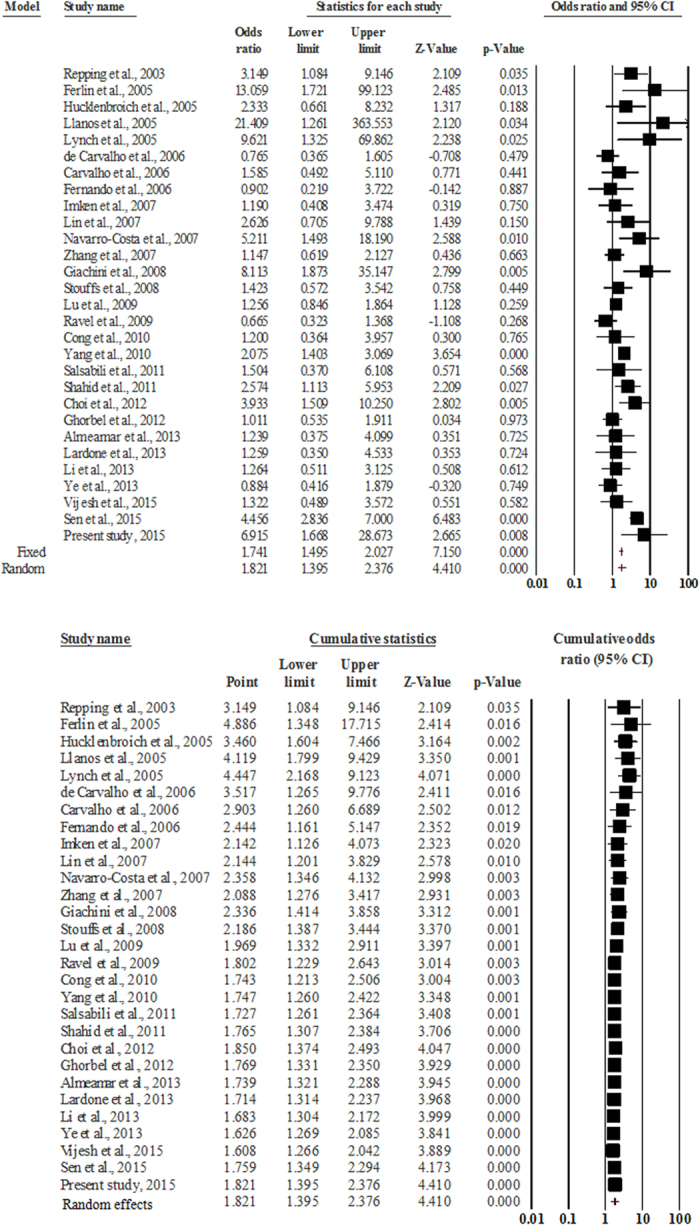
Forest plot (overall): upper panel shows the pooled estimate of all studies; lower panel shows the cumulative statistics.

Since the studies pooled in this analysis had been undertaken in different laboratories on various populations, internal heterogeneity was expected; therefore, we undertook the analysis with a priori preference for the random effects model. The pooled data showed a moderate true heterogeneity (Q-value = 68.36, df (Q) = 28, I^2^ value = 59.09, p-value = 0.00) (Table 3). Meta-analysis revealed a significant association between the gr/gr deletions and male infertility (Fixed effect model: OR = 1.741, p-value = 0.0; Random effects model: OR = 1.821, p-value = 0.0) (Fig. 3). The asymmetrical distribution of studies on the funnel plot suggested the presence of publication bias and the requirement for imputation of three additional studies (supplementary Fig. 2). However, the presence of publication bias was ruled out by the Egger’s regression intercept test (two-tailed p-value = 0.263). We performed a sensitivity analysis by removing five studies (aforementioned) based on a relatively smaller sample size (less than a hundred) in either of the comparison groups. However, no notable difference in the odds ratio, p-value, heterogeneity and publication bias was observed, ruling out the presence of sensitive studies in the pool.

**Figure 4:**
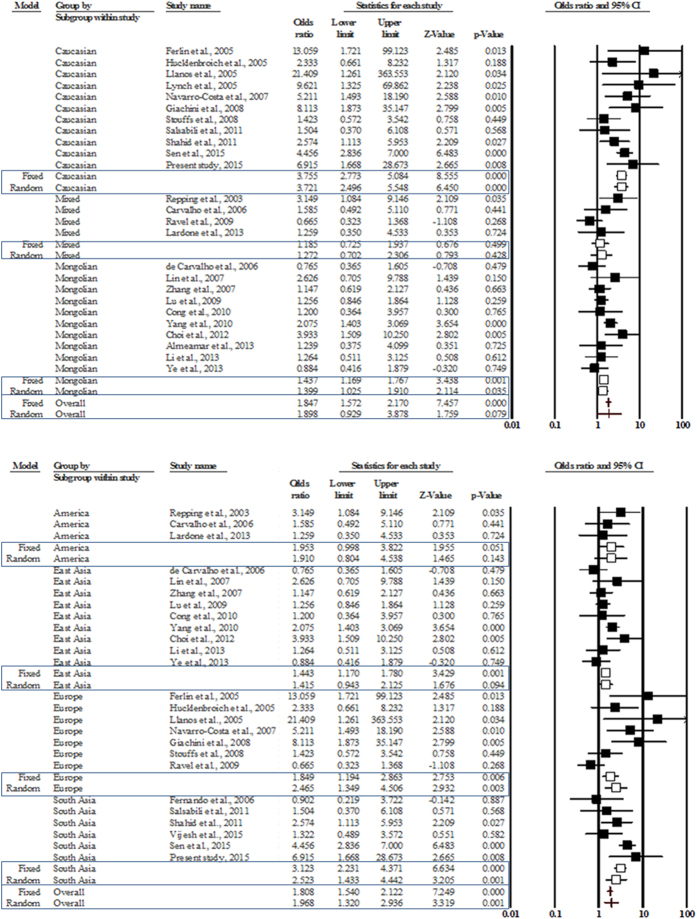
Forest plot (sub-group): upper panel shows pooled estimate on the sub-groups based on ethnicity; lower panel shows the pooled estimate on the sub-groups based on the geographic region.

Meta-analysis on the basis of ethnicity revealed that the gr/gr deletions correlate with infertility in the Caucasian and Mongolian populations, but not in the populations with mixed ethnicity (Fig. 4). Nevertheless, the association between the gr/gr deletions and male infertility in the Mongolian populations was only marginally significant (p-value = 0.035). Further, we observed a true heterogeneity of low to moderate levels in all these populations; therefore, we considered the random effects model for all further comparisons (Table 3). The Caucasian individuals with gr/gr deletions had a higher (3.7 times) risk of male infertility in comparison to those without deletions (OR = 3.721, p-value = 0.0). Furthermore, the individuals with gr/gr deletions in the Mongolian and mixed populations had 1.4 and 1.3 times higher risk of infertility, respectively, in comparison to the individuals without deletions (Mongolians: OR = 1.399, p-value = 0.035; Mixed: OR = 1.272, p-value = 0.428) (Table 3).

The region-wise meta-analysis revealed that the gr/gr deletions were significantly associated with male infertility in European (OR = 2.465, p-value = 0.003) and South Asian (OR = 2.523, p-value = 0.001) regions, but not in American (OR = 1.910, p-value = 0.143) and East Asian (OR = 1.415, p-value = 0.094) regions (Fig. 4). Only a few studies were available from Australian (n = 1), North African (n = 2), and South-East Asian (n = 1) regions; therefore, we did not take up subgroup analysis on these regions. Studies from America were homogeneous (I^2^ = 0.0, p-value = 0.51) while those from East Asia and Europe had moderate (I^2^ = 42.56, p-value = 0.08) and high (I^2^ = 71.64, p-value = 0.00) heterogeneity, respectively. European individuals with gr/gr deletions had 2.5 times higher risk of male infertility in comparison to those without deletions (OR = 2.465, p-value = 0.003). Though, the comparison between infertile and fertile groups in American and East Asian populations revealed a statistically non-significant difference, yet the individuals with gr/gr deletions in these populations had 1.9 and 1.4 times higher risk of infertility, respectively, in comparison to the individuals without deletions (Table 3).

### Gr/gr deletions correlate with low sperm count

**Figure 5:**
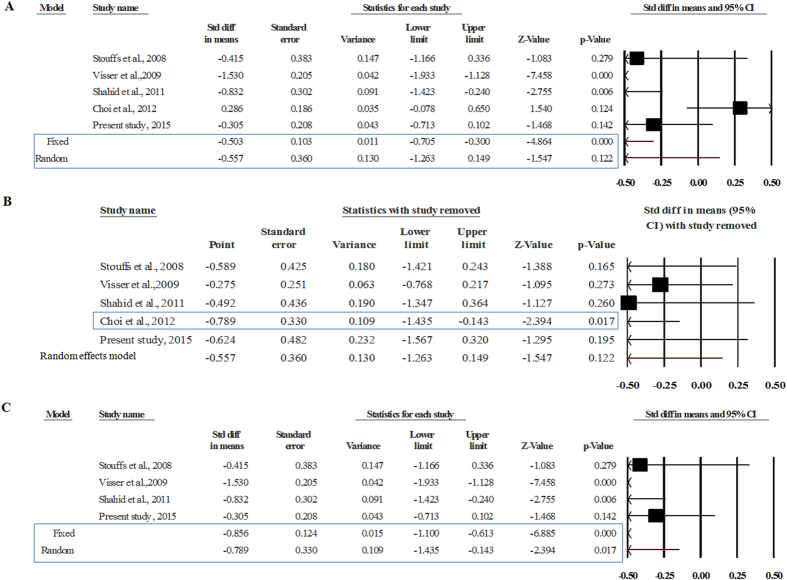
Meta-analysis comparing sperm count between gr/gr deleted and non-deleted groups: (**A**) overall meta-analysis, (**B**) sensitivity analysis showing the sensitive study (Choi *et al.*, 2012), and C) Pooled estimate after removal of the sensitive study.

In total, five studies provided adequate data for undertaking a meta-analysis on sperm count. Four of these reported an inverse correlation between gr/gr deletions and sperm count; two with a statistically significant p value [Visser *et al.* (2009)^21^ and Shahid *et al.* (2011)^30^] and two with a non-significant p value [Stouffs *et al.* (2008)^64^ and the present study (2015)] (Fig. 5A). Interestingly, Choi *et al.* (2012)^68^ observed a direct, but non-significant correlation between the gr/gr deletions and sperm count (Fig. 5A). We divided the cases/controls into two sub-groups, gr/gr deleted and non-deleted. The heterogeneity test revealed the presence of a high level of true heterogeneity (I^2^ = 91.16, p-value = 0.0). Therefore, in all further analyses, we considered the random effects model for the inference. The average sperm count in the deletion group was low in comparison to the no-deletion group (Gr/gr deleted = 34.85 million/ml; gr/gr non-deleted = 46.53). Nevertheless, the average sperm count in both the groups was within the normal range as per the WHO 2010 criteria.

We performed a meta-analysis on the data extracted from five studies detailed above. Overall estimates showed a non-significant association between the gr/gr deletions and sperm count (MD = −0.557, SE = 0.36, p-value = 0.122) (Fig. 5A). Further, the sensitivity analysis revealed that Choi *et al.* (2012)^68^ had a significant impact on the results of meta-analysis (Fig. 5B). Choi *et al.* (2012) reported a direct correlation between the gr/gr deletions and sperm count. Exclusion of this study revealed a highly significant inverse correlation between the gr/gr deletions and sperm count (MD = −0.789, SE = 0.33, Z = −2.394, p-value = 0.017) (Fig. 5C). We observed a symmetrical distribution of the studies on funnel plot, which suggested the absence of publication bias. Further, the Egger’s regression intercept test also confirmed the absence of publication bias (2-tailed p-value = 0.766).

### TSA confirms a significant correlation of gr/gr deletions with male-infertility

**Figure 6:**
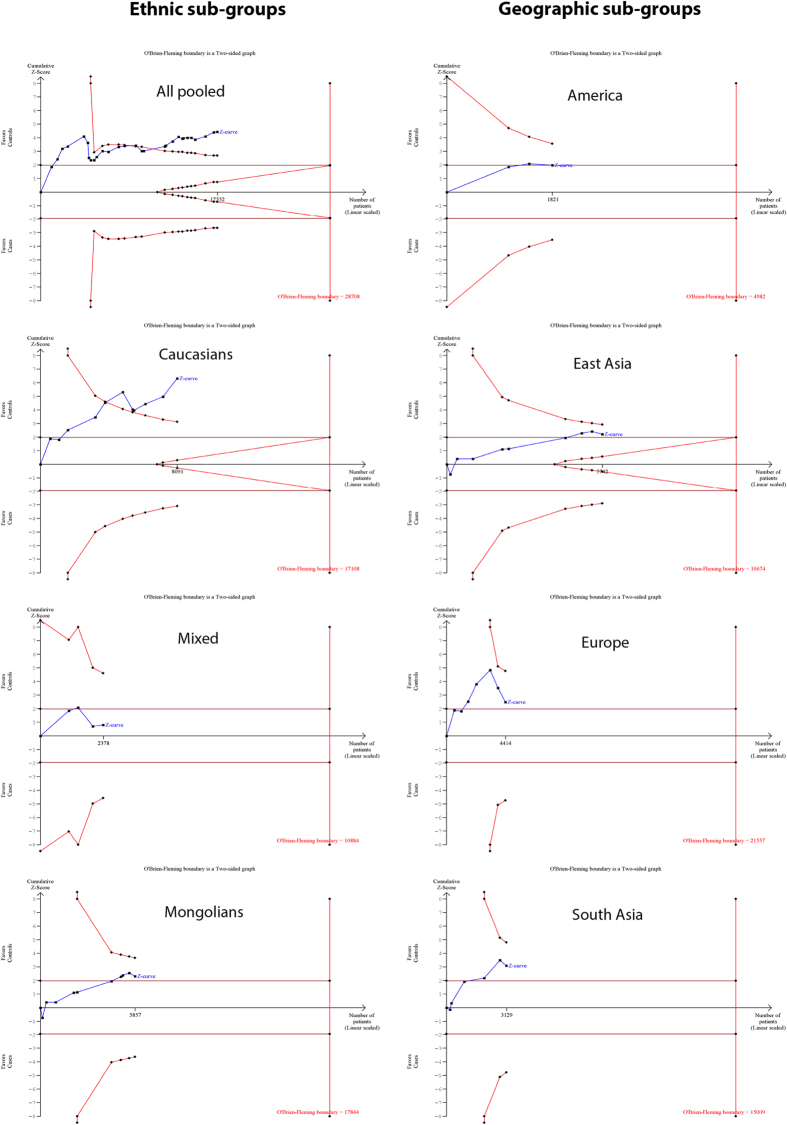
Trial sequential analysis on all pooled studies, ethnicity-wise and geographic region-wise.

The results of meta-analysis were further confirmed by trial sequential analyses. Outcomes of the trial sequential analysis were concordant with the results of the traditional meta-analysis, suggesting that the gr/gr deletions are significantly associated with male-infertility risk. In the TSA graph for all studies, the blue line (cumulative Z-score) crossed the inwards sloping red line (trial sequential boundary), providing the evidence of the effect of gr/gr deletions on male infertility risk (Fig. 6). Nevertheless, TSA suggested the requirement of some additional trials to achieve 80% study power, as the required information (sample) size calculated by the TSA was much higher (28708) than the total size pooled in this study (17552) (Fig. 6).

The TSA results for subgroups based on ethnicity were concordant with those of the conventional meta-analyses for Caucasian and mixed populations, but not for Mongolian populations. TSA suggested that the gr/gr deletions are a risk factor in the Caucasian individuals, but not in the Mongolians and mixed populations (Fig. 6). The blue line crossed the trial sequential monitoring boundary only in the case of Caucasian populations. While the conventional meta-analysis suggested a marginally significant association in the Mongolian populations (p = 0.035), TSA ruled out the association between gr/gr deletions and male infertility in these populations. Nonetheless, all TSA graphs suggested the requirement of some additional trials (samples) to achieve 80% study power.

The TSA results for subgroups based on the geographic location were consistent with those of the conventional meta-analysis for America and East Asia, but not for Europe and South Asia (Fig. 6). The blue line (cumulative Z score) in the TSA graph crossed the conventional significance boundary, but failed to cross the trial sequential boundaries for studies from Europe and South Asia (Fig. 6). Thus, the TSA indicated the presence of bias in the outcomes of meta-analysis for European and South Asian groups. Nevertheless, TSA also suggested the requirement of additional trials to reach 80% study power (Fig. 6).

## Discussion

The present study was conducted on an untested Indian population with clearly defined cases and controls to analyze the frequencies of complete and partial deletions in the AZFc region of the Y-chromosome. The complete AZFc deletions were seen in the cases only, which goes well with the already established cause and effect relationship between these deletions and spermatogenic failure^43^,^44^. Therefore, further discussion in this paper focuses only on the partial deletions. Among the partial deletions, the most frequent and significant were the gr/gr deletions, which significantly increased the risk of infertility in the study population. The literature suggests that R and H are the major haplotypes in the north Indian populations^75^. Since R and H haplotypes do not have the gr/gr deletions fixed, the above observation complies with the hypothesis that the gr/gr deletions are a risk factor outside a deletion fixed haplotype. Further, the gr/gr deletions were observed both in the cases and controls, which suggested their persistence in the population as polymorphisms and their propagation (from father to son) through some certain mechanism that balances between its selection and the rate of homologous rearrangements. Although we also observed other partial deletions, but their frequencies were very low and they did not correlate with infertility.

Since the first report of the gr/gr deletions^14^, more than 12000 men have been studied across the world for the pathogenic effects of these deletions on male fertility. However, the association of gr/gr deletions with male infertility remained unclear, raising doubts about their clinical utility^25^,^48^,^64^,^76^. This discrepancy has arisen due to the differences in study designs, methods of association analysis (cohort versus case-control), and ethnic variations^77^. A number of case-control studies on gr/gr deletions reported their significant correlation with male infertility^14^,^18^,^19^,^54^,^78^ while many others reported a lack of such an association^23^,^24^,^25^,^50^,^55^,^56^,^58^,^59^,^60^,^62^,^64^,^65^. Amid the controversies, two meta-analyses have been published, both suggesting that the gr/gr deletions increase the risk of infertility [Tuttelmann *et al.*, (2007)^79^: OR = 1.81, p-value < 0.00001; Stouffs *et al.*, (2011)^46^: OR = 1.76, p < 0.001]. However, none of these meta-analyses was subjected to further critical analysis. A number of the recent studies have generated data on a few other populations. Therefore, we performed a meta-analysis on all eligible studies and found that the gr/gr deletions are significantly associated with an increased risk of male infertility (Fixed effect model: OR = 1.741, p-value = 0.000; Random effects model: OR = 1.821, p-value = 0.000). As meta-analysis is prone to systematic and random errors, we also performed a trial sequential analysis (TSA) to confirm the results. TSA suggested that the gr/gr deletions are a risk factor for male infertility; however, the requirement of additional trials to achieve 80% power is warranted.

Sub-group analysis based on ethnicity suggested a significant association of the gr/gr deletions with male infertility risk in the Caucasian and Mongolian populations, but not in those with mixed ethnicity. However, TSA suggested that the gr/gr deletions were not a significant risk factor in the case of Mongolian populations as the conventional meta-analysis had suggested. Further, meta-analysis based on the geographic region suggested a significant association between the gr/gr deletions and male infertility in Europe and South Asia, but not in other geographic regions. Since Europe is largely inhabited by the Caucasian people, similar results in Caucasians and Europe suggest a significant impact of these deletions on male fertility in Caucasians. The lack of a significant association in TSA on Europeans may be due to a relatively smaller number of studies (four Caucasian populations were not European)^19^,^30^,^67^,^74^ and higher (I^2^ = 71.64%) heterogeneity in the European sub-group in comparison to the Caucasian sub-group (I^2^ = 26.83%). Stouffs *et al.*, (2011)^46^ also had reported a significant association of the gr/gr deletions with male infertility risk in the European region (OR = 3.05, 95% CI = 1.33–7.03). However, a critical analysis using TSA suggested that a higher number of studies would be required to confidently establish the correlation in the European populations. Despite the pooling of data from a relatively higher number of studies (29 versus 18 studies) and a larger sample size (10,948 cases and 6604 controls versus 6388 cases and 6011 controls) in comparison to Stouffs *et al.*, (2011)^46^, our analysis suggests the need of further studies from the European and South Asian regions.

In a typical case-control study, the infertile individuals are classified as cases and fertile individuals as controls, without taking sperm count into consideration. It is important to note that it has some limitations, as fertility and normozoospermia are not synonymous^21^. The very phenotypic dichotomization of the subjects into distinct groups while studying the continuous and quantitative traits, such as spermatogenesis, makes it difficult to understand the effects imparted by a single locus in a case-control design. Inclusion of a cohort analysis comparing sperm count between groups based on the deletion status may unveil the impact of a deletion on sperm count; which may otherwise be obscured in a case-control design. Therefore, we also undertook a cohort analysis to quantify the impact of the gr/gr deletions on sperm count. A relatively low sperm count in the deletion group suggested an adverse effect of the gr/gr deletions on sperm count. This may have an impact on fertility as the latter falls significantly disproportionately with the decrease in sperm count^30^. Similar to the above, Shahid *et al.*, (2011)^30^ observed that the individuals with gr/gr deletions had a relatively lesser sperm count (Oligozoospermic group: sperm count range = 5–16 million/ml) in comparison to those without deletions (Oligozoospermic group: sperm count range = 7–23 million/ml). However, we compared the gr/gr deletions across a wider range of sperm count. Visser *et al.*, (2009)^21^ also revealed that the individuals with gr/gr deletions had a significantly lesser sperm count (34 million/ml vs 53 million/ml, p-value = 0.017) in comparison to those without deletions. Further, Sato *et al.*, (2014)^31^ concluded that the gr/gr deletions are associated with low semen quality. We also undertook a meta-analysis on the studies providing sperm count data for subjects with and without gr/gr deletions. After exclusion of a sensitive study, this analysis suggested a significant correlation between the gr/gr deletions and low sperm count.

In conclusion, the gr/gr deletions significantly increased the risk of infertility in the study population. Meta-analysis also suggested that the gr/gr deletions are significantly associated with the risk of male infertility, especially in the Caucasian populations. A cohort analysis suggested that the individuals with deletions had a relatively lesser average sperm count in comparison to the group without deletions, which was supported by a meta-analysis on gr/gr deletions and sperm count. The effect on sperm count, even if little, imparted by the deletions could make an individual prone to further deterioration of spermatogenesis due to a variety of other risk factors, such as life-style, nutrition, epigenetic and other genetic variations that affect sperm count. In addition to identifying the infertility risk due to Y-partial deletions, our study reports at least one factor that could explain the inter-study variations with respect to the association of Y-deletions with male infertility. On the basis of our dual approach of data analysis, we strongly recommend the comparison of average sperm count between the groups stratified on the basis of deletion status. Re-arrangements due to the deletions and propensity to further unclassified/unknown rearrangements could also contribute to significant variations across the studies. For example, our observation of the loss of sY1161 in two individuals and sY1201 in four individuals may indicate the loss of Y-chromosome parts beyond AZFc. Variations across the studies could be further explained by investigation of the effect of deletions on gene expression and protein function. TSA suggested inclusion of more studies in the meta-analysis; therefore, further analysis of Y-partial deletions in various populations is encouraged. Further, sub-group analysis suggested that ethnicity based grouping provides more realistic results than grouping based on the geographic location. In a nutshell, the gr/gr deletions appear to strongly affect sperm count and fertility, and could be screened in patients in a clinical setting for understanding the causes of infertility, loss of sperm count and for offering counseling to the patients. Other partial deletions (b2/b3 and b1/b3) do not appear to be important, but further research may be required to firmly rule out their clinical importance.

## Additional Information

**How to cite this article**: Bansal, S. K. *et al.* Gr/gr deletions on Y-chromosome correlate with male infertility: an original study, meta-analyses, and trial sequential analyses. *Sci. Rep.* **6**, 19798; doi: 10.1038/srep19798 (2016).

## Supplementary Material

Supplementary Information

## Figures and Tables

**Table 1:** The frequency of the AZFc deletions in controls and various case sub-groups.

		Partial deletions distribution (%)				Complete AFZc deletion (%)
Subjects	N	gr/gr	b1/b3	b2/b3	Total (%)	b2/b4
**All cases**	**822**	**48 (5.84)**	**1 (0.12)**	**2 (0.24)**	**51 (6.20)**	**8 (0.97)**
Azoospermic	251	3 (1.20)	Nil	1 (0.40)	**4 (1.59)**	4 (1.59)
Oligozoospermic	105	7 (6.67)	Nil	Nil	**7 (6.67)**	Nil
Asthenozoospermic	34	Nil	Nil	Nil	**Nil**	Nil
Normozoospermic	203	18 (8.87)	Nil	Nil	**18 (8.87)**	Nil
Uncategorized idiopathic infertile	229	20 (8.73)	1 (0.44)	1 (0.44)	**22 (9.61)**	4 (1.75)
**Fertile controls**	**225**	**2 (0.89)**	**Nil**	**1 (0.44)**	**3 (1.33)**	**Nil**

**Table 2:** The comparison of Y-partial deletions data between cases and controls.

Partial deletions	Groups	Cases N = 822 (%)	Controls N = 225 (%)	Statistical comparison/One tailed P value*
gr/gr deletion	With deletion	48 (5.84)	2 (0.89)	0.0004
b1/b3 deletion	With deletion	1 (0.12)	0	0.77
b2/b3 deletion	With deletion	2 (0.24)	1 (0.44)	0.54

^*^Fisher’s exact probability test was used for comparison between two groups.

**Table 3:** Pooled estimate and statistical significance in various analysis groups.

	Models	OR	P-value	Q-value	I^2^ value	P-value
Pooled estimate	Fixed effect	1.741	0.000	68.36	59.04	0.00
	Random effects	1.821	**0.000**			
Ethnicity wise						
Caucasian	Fixed effect	3.755	0.000	13.67	26.83	0.19
	Random effects	3.721	**0.000**			
Mongolian	Fixed effect	1.437	0.001	13.99	35.66	0.12
	Random effects	1.399	**0.035**			
Mixed	Fixed effect	1.185	0.499	5.94	49.46	0.11
	Random effects	1.272	0.428			
Region wise						
East Asia	Fixed effect	1.443	0.001	13.93	42.56	0.08
	Random effects	1.415	0.094			
Europe	Fixed effect	1.849	0.006	21.16	71.64	0.00
	Random effects	2.465	**0.003**			
South Asia	Fixed effect	3.123	**0.000**	10.65	53.04	0.06
	Random effects	2.523	0.001			
America	Fixed effect	1.953	0.051	1.34	0.000	0.51
	Random effects	1.910	0.143			
