# Supplementary material for: Gr/gr deletions on Y-chromosome correlate with male infertility: an original study, meta-analyses, and trial sequential analyses
Source: Sci Rep. 2016 Feb 15;6:19798. doi: 10.1038/srep19798 (PMC4753437; doi:10.1038/srep19798)

Gr/gr deletions on Y-chromosome correlate with male infertility: an original study, meta-analyses, and trial sequential analyses

Sandeep Kumar Bansal[1], Deepika Jaiswal[2], Nishi Gupta[1], Kiran Singh[2], Rima Dada[3], Satyanarayan Sankhwar[4], Gopal Gupta[1], Singh Rajender[1*]

Supplementary information

Supplementary Table S1: Primer sequences and PCR conditions used for amplification.

Y-chromosome partial deletion markers	Primer set sequences Forward (F) and reverse (R) (5'-3')	Amplicon size (Base pair)	Annealing temperature	Annealing time (Sec)	Extension time (Sec)
**sY1161**	F=CGACACTTTTGGGAAGTTTCA<br>R=TTGTGTCCAGTGGTGGCTTA	377	54°C	30	30
**sY1191**	F=CCAGACGTTCTACCCTTTCG<br>R=GAGCCGAGATCCAGTTACCA	385	65°C	30	30
**sY1291**	F=TAAAAGGCAGAACTGCCAGG<br>R=GGGAGAAAAGTTCTGCAACG	527	67°C	30	30
**sY1206**	F=ATTGATCTCCTTGGTTCCCC<br>R=GACATGTGTGGCCAATTTGA	394	65°C	25	30

**sY1201**	F=CCGACTTCCACAATGGCT<br><br>R=GGGAGAAAAGTTCTGCAACG	677	66°C	30	35

*PCR conditions were- **Step 1**: Initial denaturation = 95°C (5'); **Step 2**: [Denaturation = 95°C (30''), Annealing = specific for each primer set, Extension = 72°C (specific for each primer set)] x 35 cycles; **Step 3:** Final extension = 72°C (7').

Supplementary Table S2: Plus/minus scheme used to identify the type of deletions.

Partial deletions/ STS markers	sY1161	sY1191	sY1291	sY1206	sY1201
**gr/gr deletion**	+	+	-	+	+
**b1/b3 deletion**	-	-	-	+	+
**b2/b3 deletion**	+	-	+	+	+
**b2/b4 deletion**	+	-	-	-	+

*Plus (+) and minus (-) signs indicate the presence or absence of the amplicon for each STS marker, respectively.

Supplementary Table S3: Characteristics of the studies included in the meta-analysis

Study name	Population/Region	STS markers	Methods	Inclusion criteria		Availability of sperm count data for meta-analysis
				Patients	Controls	
**Repping et al., 2003**	Mixed, USA	sY1191, sY1197, sY1291, sY142, sY1201, sY1206	STS-PCR	Non obstructive azoospermia and severe oligozoospermia	Normozoospermic/ proven fertile	NA
**Ferlin et al., 2005**	Caucasian/ northern Italy	sY1161, sY1191, sY1197, sY1201, sY1206 ,sY1258, sY1291, sY142	STS-PCR	Normal karyotype, No Yq microdeletion, idiopathic infertile	Normal karyotype, No Yq microdeletion, Normozoospermic	NA
**Hucklenbroich et al., 2005**	Germany	sY1161, sY1191, sY1197, sY1201, sY1206, sY1258, sY1291, sY142, sY143	Multiplex PCR	Normal karyotype, No known cause of male infertility, no Yq microdeletion	Clinically healthy, normal semen and hormonal parameters	T-test between gr/gr deleted cases (n=14) and controls (n=3) based on sperm concentrations revealed a significant difference (1.76±2.91 and 77.2±22.7, respectively; P <0.001)
**Llanos et al., 2005**	Barcelona	sY1161, sY1191, sY1201, sY1206, sY1291	Multiplex PCR	Normal karyotype, No AFZc complete deletion, ICSI patients	Semen donors/ proven fertile/ patients with conditions unrelated to infertility	NA
**Lynch et al., 2005**	Australian, Monash	sY1161, sY1191, sY1201, sY1206, sY1291	Multiplex PCR	Normal karyotype, idiopathic infertile	Proven fertile/normozoospermic	NA
**de Carvalho et al. 2006**	Japanese	sY1291, sY1161, sY1191, sY1201, sY1206, sY1258	Duplex PCR	Azoospermic/ infertile	Unknown fertility	NA
**Carvalho et al. 2006**	Brazilian	sY1161, sY1191, sY1206, sY1201, sY1258	Duplex PCR	Normal karyotype, idiopathic infertile, no AZFc microdeletion	Proven fertility/ presumably fertile	NA
**Fernando et al., 2006**	Sri Lankan	sY1161, sY1191, sY1201, sY1206, sY1291	Multiplex PCR	Nonobstructive azoospermia/ infertile	Normal spermatogenesis	NA
**Imken et al., 2007**	Moroccan	sY1191, sY1291	Multiplex PCR	Idiopathic infertile	Proven fertility/ normozoospermic	NA
**Lardone et al. 2007 (study excluded)**	Chilean men	sY1291, sY1161, sY1191, sY1201, sY1206, sY1258	Single PCR	Normal karyotype, no Yq microdeletion, idiopathic infertile	Proven fertility	Remark: Study was excluded because samples were used in Lardone et al., 2013.

**Lin et al., 2007**	Han- Chinese, Taiwan	sY1161, sY1191, sY1201, sY1206, sY1291	Multiplex PCR	Oligozoospermic	Proven fertility	NA
**Navarro-Costa et al., 2007**	Portuguese	sY1197, sY1192, sY1291, sY1206, sY1201, sY142	STS-PCR	Normal karyotype, idiopathic infertile, no Yq microdeletion	Proven fertile	NA
**Wu et al., 2007 (study excluded)**	Han- Chinese/East China	sY1161, sY1191, sY1197, sY1201, sY1206, sY1258, sY1291, sY1054	Multiplex PCR	Normal karyotype, idiopathic infertile	Proven fertile with normozoospermi a	Remark: Study was excluded because samples were used in Lu et al., 2009.
**Zhang et al., 2007**	Chinese	sY1161, sY1191, sY1201, sY1206, sY1291	STS-PCR	Idiopathic infertile	Proven fertility/ normozoospermi c	NA
**Giachini et al., 2008**	Italian	sY1161, sY1191, sY1197, sY1201, sY1206, sY1291, sY142	STS-PCR	Normal karyotype, no Yq microdeletion, idiopathic infertile	Normozoosperm ic	NA
**Lu et al., 2009**	Han- Chinese/ East China	sY1161, sY1191, sY1197, sY1201, sY1206, sY1258, sY1291, sY142, sY1054	Multiplex PCR	Normal Karyotype, No Yq microdeletion, idiopathic infertile	Proven fertility	NA
**Stouffs et al., 2008**	Belgium	sY1291, sY1191, sY1197	STS-PCR	Normal karyotype, No AZFc microdeletion	Proven fertile, normozoospermi c, normal spermatogenesis	Control cohort (mean±SD)[*]<br><br>gr/gr deleted (n=7): 69.9±39.4<br><br>gr/gr non-deleted (n=271): 91.3±51.8
**Ravel et al., 2009**	France	sY1291, sY1191	STS-PCR	Normal karyotype, idiopathic infertile, no Yq microdeletion	Proven fertile/ normozoospermi c, no Yq microdeletion	T-test between gr/gr deleted cases (n=15) and controls (n=8) based on sperm concentrations revealed a significant difference (3.2±4.73 and 65.4±34.2, respectively; P <0.001)
**Visser et al., 2009**	Singapore	(sY142, sY1197, sY1191, sY1291, sY1206,sY1201)	STS-PCR	Normal karyotype, idiopathic infertile, no Yq microdeletion	No controls	Case cohort (mean±SD)[*]<br><br>gr/gr deleted (n=25): 34±15.25<br><br>gr/gr non-deleted (n=1016): 53±12.34
**Cong et al., 2010**	Chinese	sY1191, sY1291, sY1201, sY1206, sY1161, sY1197, sY1258, sY254, sY255	STS-PCR	No Yq microdeletion, idiopathic infertile	Normal fertility	Data could not be extracted because of unavailability of the full text from the Chinese journal.
**Yang et al., 2010**	Chinese	sY1191, sY1201, sY1206, sY1161, sY1054, sY1125, sY1291, sY254	STS-PCR	Normal karyotype, No Yq microdeletion, idiopathic infertile	Normozoosperm ic	NA

**Salsabili et al., 2011**	Iranian	sY1291, sY1191	Multiplex PCR	Normal karyotype, idiopathic infertile, no Yq microdeletion	Proven fertile but sperm count <20 Million/ml	NA
**Shahid et al., 2011**	North Indian, New Delhi	sY1161, sY1191, sY1197, sY1201, sY1206, sY1258, sY1291, sY142	Simplex PCR	Normal karyotype, idiopathic infertile, no AZFc microdeletion	Proven fertile men with normozoospermia	Case cohort (mean±SD)*<br>gr/gr deleted (n=12): 10.75±3.2<br>gr/gr non-deleted (n=170): 13±2.67
**Choi et al., 2012**	South Korean	sY1191, sY1291, sY1206	Multiplex PCR	Idiopathic infertile	Normozoospermic	Case cohort (mean±SD)*<br>gr/gr deleted (n=32): 5.4±14.8<br>gr/gr non-deleted (n=318): 2.9±7.9
**Ghorbel et al., 2012**	Tunisian	sY1191, sY1291	Multiplex PCR	Idiopathic infertile	Proven fertile men	NA
**Almeamar et al., 2013**	Malaysian	sY1191, sY1291	Duplex PCR	Idiopathic infertile	Proven fertile men	NA
**Lardone et al., 2013**	Chilean	sY1161, sY1191, sY1197, sY1201, sY1206, sY1258, sY1291	Single PCR	Idiopathic infertile	Proven fertility/ normozoospermic/ non-obstructive azoospermia with normal hormonal parameters	NA
**Li et al., 2013**	South Chinese	Seven AZFc STS markers	STS-PCR	Patients with spermatogenic impairments	Normozoospermic	NA
**Ye et al., 2013**	Yi population, Yunnan Province, China	NA	STS-PCR	Idiopathic infertile	Fertile men	NA
**Vijesh et al., 2015**	South India	sY1191, sY1291, sY1201, sY1161, sY1206, sY1054, and sY1197	STS-PCR	Idiopathic infertile, No Yq microdeletion	Normal fertile men	NA
**Sen et al., 2015**	Maharashtra, South India	sY1161, sY1191, sY1206, sY1291	STS-PCR	Normal karyotype, Idiopathic infertile, No Yq microdeletion	Normozoospermic with proven fertility status	NA
**Present study, 2015**	North Indian, New Delhi	sY1161, sY1191, sY1201, sY1206, sY1291	STS-PCR	Normal karyotype, idiopathic infertile	Proven fertile	Case cohort (mean±SD)*<br>gr/gr deleted (n=25): 54.20±57.45<br>gr/gr non-deleted (n=317): 72.49±60.06

NA= Data could not be extracted. *Indicates sperm count (million/ml) in case/control cohorts. This data were used for performing the meta-analysis on sperm count.

Supplementary Table S4: Genotype details of the studies included in the meta-analysis.

Study name	Redefined geographic region	Ethnicity	Cases			Controls		
			Total (n)	Deleted (n)	Non-deleted (n)	Total (n)	Deleted (n)	Non-deleted (n)
**Repping et al., 2003**	America	Mixed	708	24	684	363	4	359
**Ferlin et al., 2005**	Europe	Caucasian	337	16	321	263	1	262
**Hucklenbroich et al., 2005**	Europe	Caucasian	348	14	334	170	3	167
**Llanos et al., 2005**	Europe	Caucasian	283	12	271	232	0	232
**Lynch et al., 2005**	Australia	Caucasian	1387	55	1332	234	1	233
**de Carvalho et al., 2006**	East Asia	Mongolian	78	22	56	56	19	37
**Carvalho et al., 2006**	America	Mixed	110	5	105	240	7	233
**Fernando et al., 2006**	South Asia	Dravidian	96	4	92	87	4	83
**Imken et al., 2007**	North Africa	Nigro-Caucasian	149	7	142	176	7	169
**Lin et al., 2007**	East Asia	Mongolian	142	10	132	107	3	104
**Navarro-Costa et al., 2007**	Europe	Caucasian	300	15	285	300	3	297
**Zhang et al., 2007**	East Asia	Mongolian	296	24	272	280	20	260
**Giachini et al.,**	Europe	Caucasian	556	18	538	487	2	485

**2008**								
**Lu et al., 2009**	East Asia	Mongolian	711	89	622	391	40	351
**Stouffs et al., 2008**	Europe	Caucasian	187	8	179	394	12	382
**Ravel et al., 2009**	Europe	Mixed	364	18	346	193	14	179
**Cong et al., 2010**	East Asia	Mongolian	160	10	150	76	4	72
**Yang et al., 2010**	East Asia	Mongolian	1426	138	1288	672	33	639
**Salsabili et al., 2011**	South Asia	Caucasian	29	4	25	52	5	47
**Shahid et al., 2011**	South Asia	Caucasian	418	30	388	240	7	233
**Choi et al., 2012**	East Asia	Mongolian	377	32	345	217	5	212
**Ghorbel et al., 2012**	North Africa	Nigro-Caucasian	261	34	227	124	16	108
**Almeamar et al., 2013**	South-East Asia	Mongolian	52	6	46	63	6	57
**Lardone et al., 2013**	America	Mixed	218	6	212	182	4	178
**Li et al., 2013**	East Asia	Mongolian	186	11	175	190	9	181
**Ye et al., 2013**	East Asia	Mongolian	224	17	207	153	13	140
**Vijesh et al., 2015**	South Asia	Dravidian	208	13	195	125	6	119
**Sen et al., 2015**	South Asia	Caucasian	515	144	371	312	25	287
**Present study, 2015**	South Asia	Caucasian	822	48	774	225	2	223

Supplementary Figure S1: Pictorial representation of the location and orientation of STS markers used for deletion screening.

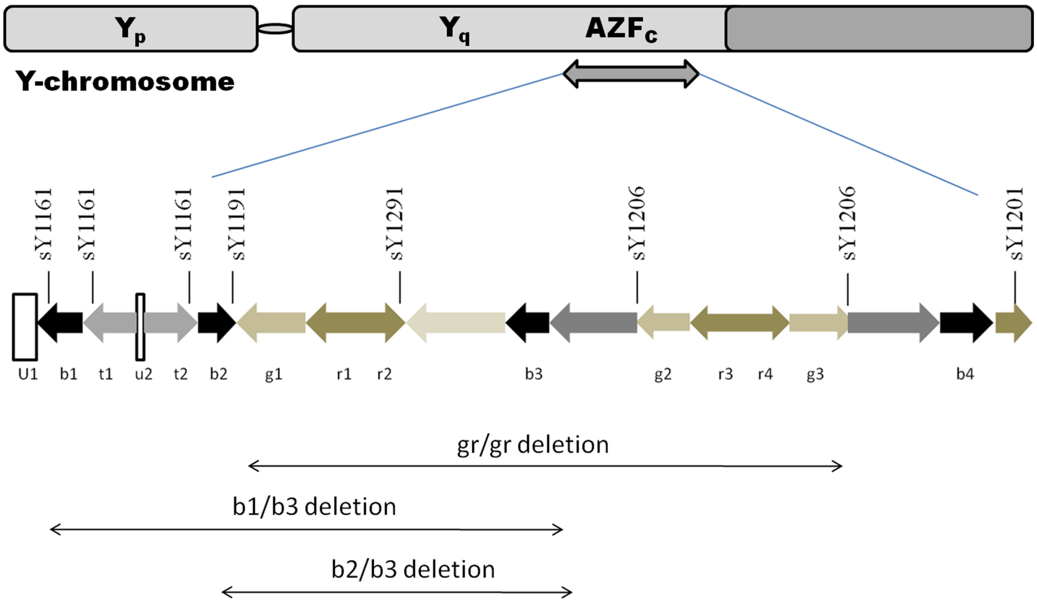

Supplementary Figure S2: Meta-analysis-funnel plot: upper panel shows the asymmetric distribution of the studies; lower panel shows the imputation of studies required for symmetry of the plot.

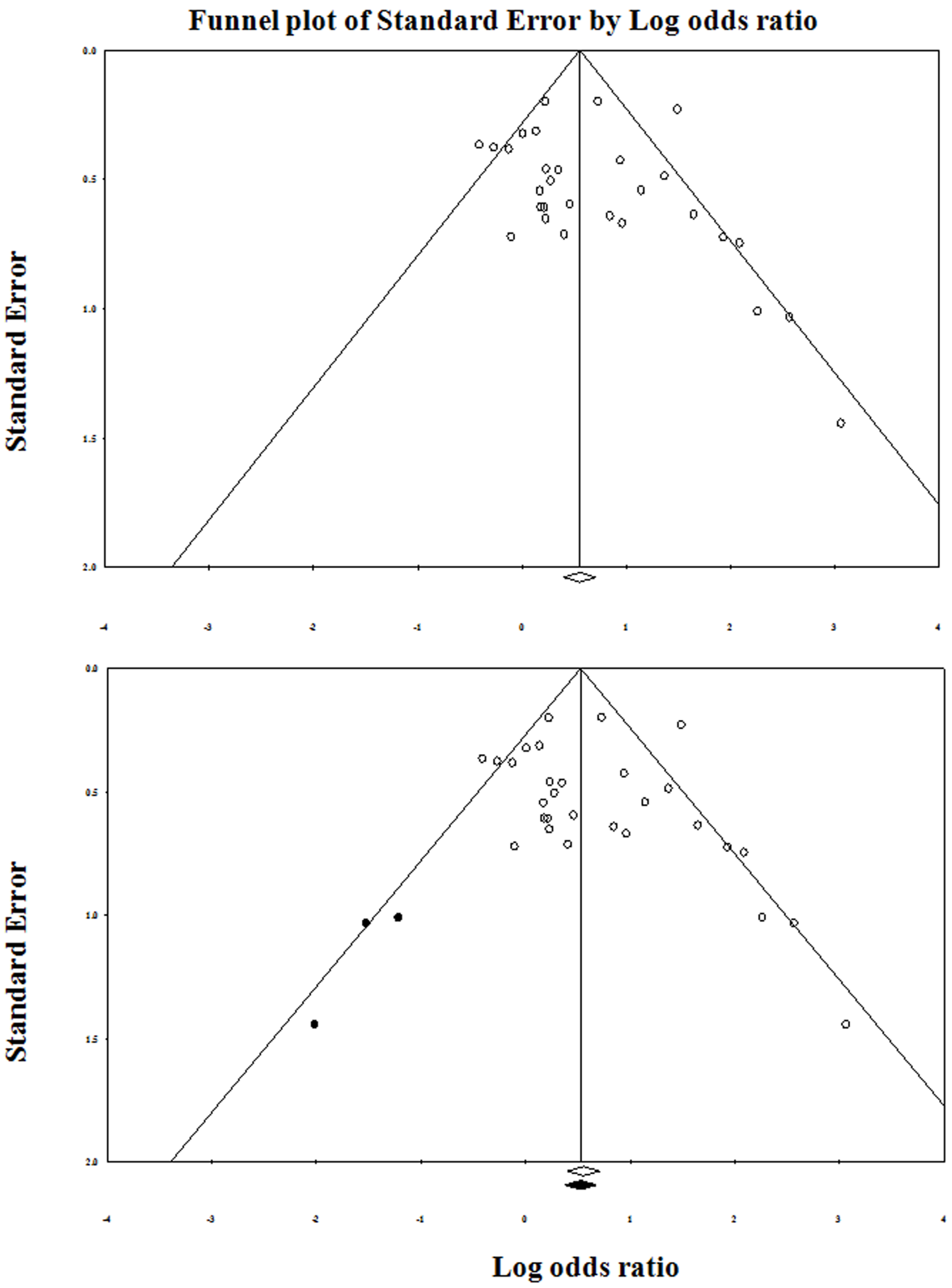

Supplement: Supplementary Information [file srep19798-s1.pdf]
